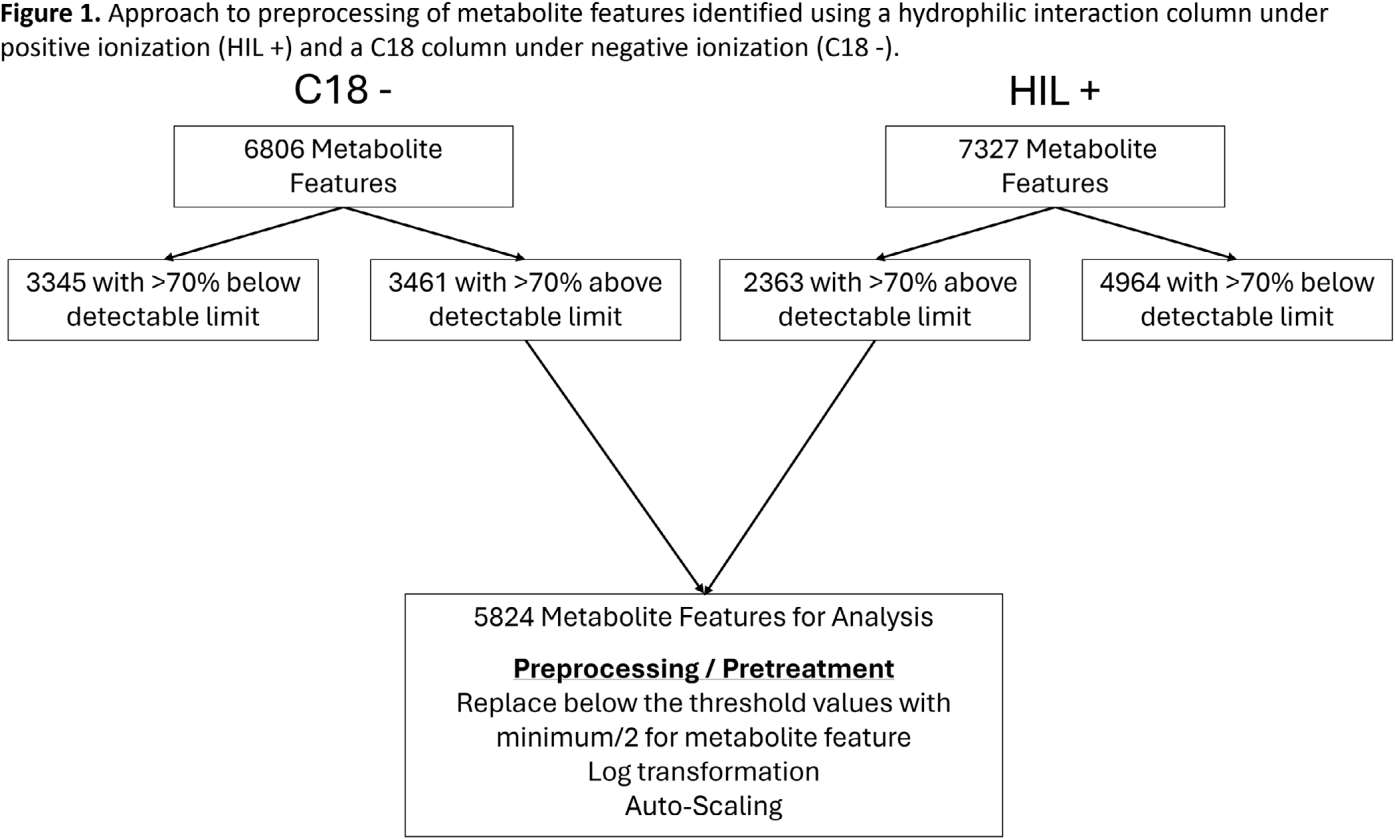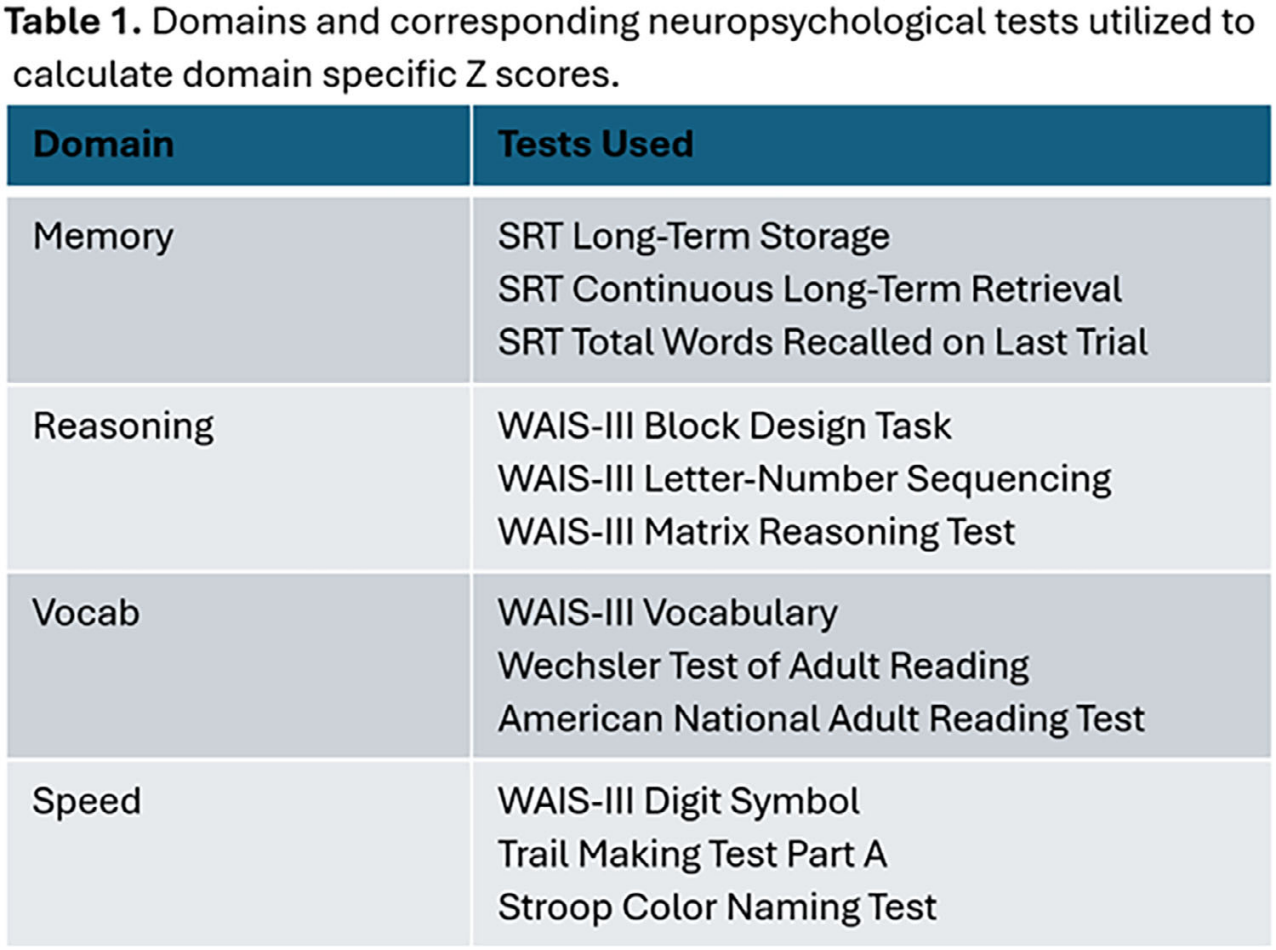# Metabolic Profiles Associated with Domain‐Specific Change in Cognition Among Cognitively Normal Adults

**DOI:** 10.1002/alz70860_106632

**Published:** 2025-12-23

**Authors:** Brandi Vollmer, Christian G Habeck, Vrinda Kalia, Gary W Miller, Yaakov Stern, Yian Gu

**Affiliations:** ^1^ Taub Institute for Research on Alzheimer's Disease and the Aging Brain, Columbia University, New York, NY, USA; ^2^ Taub Institute for Research on Alzheimer's Disease and the Aging Brain, Columbia University, New York City, NY, USA; ^3^ Mailman School of Public Health, Columbia University, New York, NY, USA; ^4^ Taub Institute for Research in Alzheimer's Disease and the Aging Brain, Columbia University, New York, NY, USA; ^5^ Columbia University Vagelos Collège of Physicians and Surgeons, New York, NY, USA

## Abstract

**Background:**

Data on metabolites associated with domain‐specific change in cognition in cognitively normal adults throughout the life course remain limited. Understanding these associations may help elucidate early metabolic alterations linked to age‐related cognitive trajectories.

**Method:**

The current study included 165 cognitively normal participants of the Reference Abilities Neural Network and the Cognitive Reserve studies of Columbia University who had metabolomic and cognitive data available. Cognition was measured at baseline and follow‐up five years later, using a neuropsychological battery that included three tests for each of the four cognitive domains including memory, reason, vocabulary, and processing speed (Table 1). Plasma collected at baseline underwent untargeted liquid chromatography and high‐resolution mass spectrometry to generate metabolic profiles. Pearson correlations between each metabolite feature and change in domain‐specific cognitive scores (delta = follow‐up z score – baseline z score) were examined. *p*‐values < 0.05 were considered statistically significant.

**Result:**

Participants had a mean age of 57.1 years (standard deviation (SD) = 16.2), 52.1% were female, 69.1% were white, 21.8% were black, and 8.1% were other or a mix of multiple races, 83.6% were non‐Hispanic and the mean education level was 15.9 years (SD = 2.4). After excluding 8,309 (Figure 1), a total of 5,824 metabolic features were analyzed, of which 300, 314, 203, and 211 were significantly correlated with change in cognitive domains of memory, reason, vocabulary, and processing speed, respectively. Notably, no metabolic features were significantly correlated with change in all 4 cognitive domains and only three metabolic features were associated with a combination of 3 domains.

**Conclusion:**

Our findings suggest unique metabolic profiles are associated with longitudinal change in different cognitive domains, indicating the heterogeneity in the biological pathways influencing different cognitive functions. Our study may provide insights into the mechanism contributing to progression from normal cognition to dementia.